# High-dose benzodiazepine use and QTc interval prolongation, a latent class analysis study

**DOI:** 10.1038/s41598-023-50489-3

**Published:** 2024-01-02

**Authors:** Lorenzo Zamboni, Igor Portoghese, Rebecca Casari, Francesca Fusina, Laura Santin, Luigi Isaia Lecca, Simone Campagnari, Silvia Carli, Thomas Zandonai, Fabio Lugoboni

**Affiliations:** 1 Unit of Addiction Medicine, Department of Internal Medicine, G.B. Rossi Hospital, Verona, Italy; 2 Department of Neuroscience, Biomedicine and Movement, University of Verona, Verona, Italy; 3 Dipartimento di Scienze Mediche e Sanità Pubblica, Università degli Studi di Cagliari, Cagliari , Italy; 4 Department of General Psychology, University of Padova, Padua, Italy; 5 Department of Pharmacology, Paediatrics and Organic Chemistry, Miguel Hernández University of Elche, Elche, Spain; 6 Addiction Science Lab at the Department of Psychology and Cognitive Science , University of Trento, Trento, Italy

**Keywords:** Psychology, Cardiology, Medical research

## Abstract

Benzodiazepine (BDZ) addiction is a widespread and multifaceted phenomenon. For many patients, especially females, the concomitant use of other drugs also increases their risk of QTc prolongation, possibly leading to complications such as seizures and even sudden death. However, the relationship between BDZ use and QTc prolongation is currently unclear. The present study aims to examine patterns of polysubstance use among a sample of Italian adults with BDZ dependence in relation with their QTc prolongation risk. We used Latent Class Analysis (LCA) on data collected from 251 inpatients of the Addiction Medicine Unit in Verona to group patients into three classes according to their substance use and their QTc prolongation risk. Results showed no significant relationship between QTc prolongation and BDZ use in any of the classes considered. We conclude that BDZs, even if used long-term and at high dosages, can be considered safe in terms of cardiovascular complications for patients.

## Introduction

Benzodiazepines (BDZs) are among the most widely used drugs worldwide. They are the most representative category of hypnotic-sedative drugs, which induce a gradual (dose-dependent) sedation of the central nervous system^1^.

Addiction to BDZs is a widespread but neglected phenomenon, although BDZ withdrawal is a serious and dangerous condition characterized by a series of typical signs and symptoms which begin to manifest within a few hours or days of discontinuing the drug, depending on the half-life of the BDZ that was taken.

BDZs are traditionally considered safe and effective in short-term treatment, but over half of the people taking BDZs are chronic users^2,3^, despite international guidelines suggesting that the duration of use should be limited to a few weeks^4^. Moreover, 15 to 44% of long-term BDZ users are addicted to the drug^5–8^. 

Surveys carried out in the 1990s in France, Germany, Italy, and the United Kingdom, showed that 3.9% of hypnotic drug users and 3.2% of anxiolytic drug users had been taking a dose exceeding the recommended one^9–11^. In Italy, about 7.5–10% of the adult population are BDZ users, with half of these being long-term users (LTU) with a diagnosis of BDZ use disorder^12^. 

The factors underlying BDZ abuse are studied and discussed in many international studies. In particular, a relevant aspect is represented by the appearance of tolerance which, in chronic users, determines an important increase in dosages in order to obtain the same effectiveness^13,14^.

Among the 23 BDZs which are currently sold in Italy, lormetazepam is the most used, followed by lorazepam and alprazolam, while consumption of other BDZs is much lower^15^.

Individual BDZs have different pharmacodynamics and pharmacokinetics, and also varying risks of abuse at high doses. Indeed, long-onset molecules seem safer than short-onset ones, since the latter have greater reinforcing effects.

In a study that highlighted the impact of high-dose benzodiazepine abuse in more than 1100 patients that were admitted to the Addiction Medicine Unit in Verona (Italy) from January 2003 to June 2018, lormetazepam was the most used BDZ, with more than 57% (630 patients) of patients using it; lorazepam followed with 11% (125), with alprazolam at 10% (111), zolpidem at 9% (102), and clonazepam at 3% (37)^16–19^.

The QT interval begins with the beginning of the QRS complex and ends with the end of the T wave and is inversely proportional to heart rate (HR). A rate-related (or corrected) QT interval (QTc) using Bazett's formula can be calculated as: QTc = QT interval/square root of the RR interval, with RR representing the distance between two successive R waves on the electrocardiogram. The upper limit of the normal QTc is 450 ms in males and 470 ms in females^20^.

Long QT syndrome (LQTS) is a myocardial repolarization disorder characterized by a prolonged QT interval on the electrocardiogram (ECG). The main symptoms in patients with LQTS include palpitations, syncope, seizures and sudden cardiac death. This syndrome can either be congenital or acquired. The acquired form is related to drug therapy and the presence of hypokalemia and hypomagnesaemia may accentuate the risk of drug-induced LQTS development. One of the main risks of LQTS is the generation of polymorphic ventricular tachycardia, i.e., a ventricular rhythm greater than 100 bpm with frequent changes in the QRS axis, in its morphology or both^21,22^.

Torsades de pointes (TdP) is a form of polymorphic ventricular tachycardia that occurs in the context of acquired or congenital prolongation of the QT interval, presenting with a heart rate between 160 and 250 bpm. These variations take the form of a progressive, sinusoidal and cyclical evolution of the QRS axis. The peaks of the QRS complexes appear to "twist" around the isoelectric line of the recording. documenting this condition tends to spontaneously regress, however multiple episodes can occur in rapid succession that can degenerate into ventricular fibrillation and sudden cardiac death. Determining the absolute and comparative risk of many drugs associated with QT interval prolongation is difficult, as most of the available data come from case reports or small series of observations. Furthermore, the incidence of QT prolongation without TdP is much higher than the incidence of TdP itself^23^.

The pathophysiological mechanism underlying drug-induced TdP is the development of abnormal depolarizations of the cell membrane in the final part of the action potential, defined as early post-depolarization (EAD), or during diastolic repolarization, termed late post-depolarization (DAD). Almost all drugs that cause LQTS cause blockage of the potassium channel, thereby inhibiting the rapid outward flow of the ion and therefore cellular repolarization^24,25^.

Furthermore, lower heart rates result in a minor potassium output from the cell during repolarization, as there are fewer repolarization events; also, the reduction of extracellular potassium increases the degree of inhibition that the drug induces on the rapid potassium current, consequently increasing the QT interval^24^.

The most common risk factor for drug-induced PTO is the female gender. In a review of the literature, of 332 patients with drug-associated Tdp, 70% were women. Compared to males, females have a longer QTc and a greater response to drugs that block the rapid potassium channel, favoring Tdp, possibly due to the effect of sex steroids on ion channel expression. Estrogen potentiates the prolongation of the QT interval induced by bradycardia and the development of arrhythmia. Conversely, androgens reduce the QT interval and make it less sensitive to drugs^26–28^.

Prior studies have found that individuals engaged in BDZ use showed patterns of polydrug use practices. For example, Votaw and colleagues (2020)^29^, applying a Latent Class Analysis (LCA) in a general population sample, identified three distinct latent classes: (1) limited polysubstance use class (approximately 54.6% of the sample), (2) binge alcohol and cannabis use class (28.5% of the sample), and (3) opioid use class (16.9% of the sample). Regarding the detox of high doses of BDZs, Zamboni et al. (2022)^30^ used LCA to identify different classes of BDZ users characterized by distinct substance combinations. LCA is a type of finite mixture model that is used to identify and describe homogenous subgroups within a heterogeneous population based on the similarity of their response patterns^31^. This method has been widely used in previous studies to examine substance use patterns^32–35^. LCA has been used in other studies to identify subgroups of substance use, misuse and addiction, e.g. to tobacco, internet etc.^36–40^; notably, it has been used to differentiate problematic alcohol users from addicted users.

Despite BDZs’ widespread use and the fact that they’re considered safe, not much is known about their potential interaction with other drugs, especially when BDZs are chronically used at high doses and in a polyuse patterns of consumption.

Since polydrug use is frequent among BDZ consumers, it appears of special interest to pay particular attention to the possible interaction between BDZs and other drugs of abuse that have a detrimental effect on cardiac function. In particular, drugs such as cocaine, amphetamines, heavy alcohol use and opioids are known to increase cardiac risk by increasing the onset of arrhythmias. A long QT interval can lead to TdP, which is a potentially fatal arrhythmia. Cocaine, for example, is known to prolong the QT interval, inducing TdP^41^. Also, heavy alcohol consumption was found to be a risk factor for a prolonged QTc interval^42^, while opioids such as methadone were found to prolong QT intervals even at conventional doses^43^.

However, to the best of our knowledge, the present study is the first to examine polysubstance use patterns among individuals who use BDZs as their primary drugs of choice, in order to identify different classes of BDZ users with distinct substance use combinations and to highlight a potential risk of QT prolongation. This approach may help to better understand the complexity and diversity in patterns of BDZ use and their associated health implications, such as for cardiovascular risk, thus facilitating the development of effective interventions.

In addition, this is the first study who presents a specific sample, like BDZ high-dosage users, in relation to QT prolongation risk.

In this study, all patients had been treated using the Verona Detox Approach with Flumazenil, a slow subcutaneous infusion of flumazenil (1 mg/die) for 7 days^44^.

## Methods

### Design of the study

Our study used a cross-sectional correlational design in investigating (1) the relationship among the variables, and (2) the classification of patients in classes. Inclusion criteria were: BDZ addiction (APA, 2013), data being collected from January 2015 to January 2020, signature of the informed consent, being of working age (18–70 years old).

All experimental protocols were approved by the ethics committee of the Verona University Hospital (approval code 683CESC).

### Measures

Participants’ data were collected considering demographics such as gender, age, age of first use, education, marital status, and employment status. The type of BDZs (anxiolytics and hypnotics) and misuse duration were considered by converting doses to diazepam equivalents (DDDE, mg) (Drug and Alcohol Services South Australia, 2014) and calculating the mean diazepam dose/day. Participants’ history of drug addiction and current drug use were assessed, considering heroin, cocaine, THC (cannabis/cannabinoids), and alcohol. To better quantify these variables, we assigned the following: (0) no drugs/alcohol used in the past 12 months, (1) previous history of drug/alcohol addiction/ addicted.

Body mass index (BMI) was calculated according to the following formula:

$${\text{BMI }}\left( {{\text{kg}}/{\text{m}}^{{2}} } \right)\, = \,{\text{weight }}\left( {{\text{kg}}} \right)/{\text{height}}^{{2}} \left( {{\text{m}}^{{2}} } \right).$$

Furthermore, liver function data (GGT, AST and ALT levels) were collected. We considered these aspects because our sample could present subjects with alcohol use disorder in comorbidities to BDZ addiction^45^.

Information on these variables was mainly obtained from medical records, from which data regarding the concomitant use of drugs that potentially influence QT length were also collected. DDDE data were based on self-report.

### Electrocardiographic measurements and analysis

The electrocardiogram was measured with a 12-channel, 12-lead electrocardiogram monitor (Marquette MAC-1200; GE Healthcare, Milwaukee, WI) over at least 10 s, by using a sampling frequency of 250 Hz. Digital records of electrocardiograms were processed by interactive software (QT Guard; GE Marquette Medical Systems, Milwaukee, WI), which detects QRS onset and T-wave offset to manually determine the maximum (QTmax) and minimum (Qtmin) QT interval from 12 leads*.*

All patients underwent an ECG upon admission, and the QT-interval was manually measured by the same cardiologist throughout the study. The formula used to compute the corrected QT-interval (QTc) was Bazett’s formula. QTc prolongation was defined as a value greater than 450 ms for men and 470 ms for women, while borderline values are considered between 430 and 450 ms for males and between 450 and 470 ms for females. Normal values are considered < 430 for males and < 450 for females^20^.

Finally, the use of prescribed drugs known to potentially prolong QT interval was also considered in our analysis. In detail, the concomitant use of prescribed drugs such as antipsychotics, antibiotics, antihistamines, and anticancer drugs, were collected from patients’ medical records. Then, QT-prolonging drugs were detected in accordance with the online database CredibleMeds^®^ (https://crediblemeds.org/research-scientists), which has been widely used in similar studies. The CredibleMeds list of QT-prolonging drugs is composed by four categories, as follows: 1. Known risk of TdP, 2. Possible risk of TdP, 3. Conditional risk of TdP, and 4. Drugs to avoid in congenital LQTS^41,46,47^.

### Data analysis

We calculated the absolute number and frequency of categorical variables, and central tendency and dispersion (mean and sd) for parametric variables.

We compared mean QTc levels between the two subgroups of drug assumption (patients taking/not taking drugs known to potentially increase the QTc interval), and between the two subgroups of occupational status (employed and unemployed/inactive) by means of a t-test for parametric variables.

We tested the correlations between the variables of interest with Pearson’s r coefficient. We tested the association between QTc length (dependent variable) and predictors such as the use of drugs that potentially prolong the QTc interval, the dosage of BDZs (expressed as diazepam equivalent), the concomitant presence of another addiction (different from BDZs), the duration of the continuous BDZ assumption, the age at the first assumption, the presence of a diagnosed psychiatric disorder, and employment status, by using a multivariable linear regression model that was adjusted for age and gender.

Then, we implemented Latent Class Analysis (LCA) considering age, age of first use, continuative use (in months), type of BDZ **(**anxiolytics and hypnotics), clinical range in corrected QTc intervals, history of addiction (alcohol, THC, cocaine, and heroin) in our models. LCA including one to six latent classes was estimated by employing the robust maximum-likelihood estimator (MLR) in Mplus 7. We performed LCA using 5000 random sets of start values and 1000 iterations, retaining the 500 best solutions for final stage optimization^48,49^

In selecting how many classes should be retained, we considered different information criteria (IC)-based fit statistics^50^: (a) the Bayesian Information Criterion (BIC)^51^; (b) the Akaike Information Criterion (AIC)^52^; (c) the Constant AIC (CAIC)^53^; (d) the Sample Adjusted Bayesian information criterion (SABIC); € the Approximate Weight of Evidence Criterion (AWE), and (f) the bootstrapped likelihood ratio (BLRT)^54^. The BLRT is a test that compares the improvement between K-class model with a K-1 class model, providing a statistical support (p-values) for the inclusion of one more class. Furthermore, we considered the Bayes Factor (BF) for pairwise comparison of fit between two neighboring class models. The cut-offs considered are: 1 < BF < 3 indicates “weak” support for the model with less classes, 3 < BF < 10 indicates “moderate” support, and BF > 10 indicates “strong” support^55^.

Finally, we considered the entropy that is a measure of models’ accuracy in classifying individuals into their most likely class. Entropy values range from 0 to 1, with scores closer to 1 indicating better fit of the data into the prescribed class structure. According to Nylund and colleagues (2007)^56^, the optimal class solution would have the lowest BIC values, lowest AIC values, lowest CAIC values, lowest SABIC values, a significant BLRT p-value, relatively higher entropy values, and conceptual and interpretive meaning.

An additional procedure in inspecting ICs was to consider “elbow plots”, that provide a graphical representation of improvements related with additional classes^57^. More specifically, the optimal number of classes should be the value at which the slope flattens, plus and minus a class.

Then, we analyzed the associations between the identified classes and liver function (GGT, AST and ALT levels). In this sense, the consideration of GGT, AST and ALT levels as outcomes should not qualitatively change the classes^49^. The DU3STEP method in MPlus^58^ was used.

### Informed consent

All procedures followed were in accordance with the ethical standards of the responsible committee on human experimentation (institutional and national) and with the Helsinki Declaration of 1975, as revised in 2000. Informed consent was obtained from all patients for being included in the study.

## Results

### Participant characteristics

From the starting 585 individuals, 430 met the inclusion criteria. Of those, 214 were removed because of missing data (> 5%) about relevant variables to this study. The final sample comprised 216 individuals (for more details about the inclusion criteria, see the supplementary Fig. 1s).

As shown in Table 1, 51.4% of the sample was female and the mean age was 47.61 (SD ± 10.61) years. Regarding employment, 55.6% were employed. The characteristics of the sample are summarized in Table 1.

**Table 1:** Descriptive characteristics of the sample.

	n	%	M	SD
Gender				
Male	105	48.6		
Female	111	51.4		
Age (years)			47.61	10.61
Employment				
Yes	120	55.6		
No	96	44.4		
Age of first BDZ use (years)			31.18	10.17
Continuous use of BDZs (months)			129.0	96.7
Heroin				
No	168	77.8%		
Former/current	48	22.2%		
Cocaine				
No	146	67.6%		
Former/current	70	32.4%		
THC				
No	163	75.5%		
Former/current	53	24.5%		
Alcohol				
No	127	58.8%		
Former/current	89	41.2%		
Psychiatric disorders				
No	178	83.2%		
Yes	36	16.8%		
Other addictions				
No	49	22.7%		
Yes	167	77.3%		
Bilirubin levels				
Low	213	98.6%		
High	3	1.4%		
ALT levels				
Normal	178	82.4%		
High	38	17.6%		
AST levels				
Normal	184	85.2%		
High	24	14.8%		
GGT levels				
Normal	180	83.3%		
High	36	16.7%		
Prescribed QT related drug				
Yes	103	47.7%		
No	113	52.3%		
QTc levels				
Normal	158	73.1%		
Borderline	37	17.1%		
Prolonged	21	9.7%		
DDDE (mg)			363	775

*ALT* alanine aminotransferase, *AST* aspartate aminotransferase, *BDZ* benzodiazepine, *DDDE* diazepam equivalents, *GGT* gamma-glutamyltransferase, *M* mean, *QTc* interval prolongation, *SD* standard deviation, *THC* tetrahydrocannabinol.

The comparison of the QTc values between the two employment classes (unemployed/inactive vs employed) did not show any significant difference (QTc = 429.33, sd = 22.85 vs QTc = 425.75, sd = 25.33; t = 1.192, df = 214, p = 0.282), as well as we did not find any significant difference of the QTc length when comparing the classes of assumption of other drugs known to potentially prolong the QTc interval (QTc = 427.59, sd = 24.82 for consumers *vs* QTc = 427.12, sd = 23.84 for not-consumers; t = 0.144, df = 214, p = 0.886) (Table 2).

**Table 2:** Comparison of QTc values by employment status and assumption of other drugs known to potentially prolong the QTc interval.

		Mean (sd)	t	df	P
Employment status	Employed	425.75 (25.33)	1.192	214	0.282
	Unemployed/inactive	429.33 (22.85)			
QT drugs assumption	Yes	427.59 (24.82)	0.144	214	0.886
	No	427.12 (23.84)			

We found a significant but low correlation between QTc and age (r = 0.176, p = 0.01). No other significant correlations were found between the variables of interest. The results of the multiple linear regression model showed that QTc length is positively associated with age (adjusted beta coefficient = 0.27; p = 0.003), and being female (adjusted beta coefficient = 0.164; p = 0.02), while the other predictors did not show a significant influence on the dependent variable (Table 3). The selected predictors explained 10% of the variance of the QTc length.

**Table 3:** Regression model for QTc.

Predictors	Adjusted beta coeff	SE	P
Constant		8.466	0.000
QT drugs	− 0.007	3.337	0.921
equivalent dose of diazepam (mg)	− 0.074	0.002	0.286
Age at first assumption	− 0.095	0.199	0.252
Duration of continuous assumption (in months)	− 0.114	0.020	0.137
Employment status	− 0.061	3.390	0.381
Psychiatric disease	− 0.047	4.529	0.502
Multiple addiction	− 0.047	4.108	0.505
Age (years)	0.265	0.205	0.003
Gender	0.164	3.423	0.021

Multiple linear regression model predicting QTc duration by predictors QT drugs (assumption of drugs known to potentially influence QT length – yes/no), equivalent dose of diazepam (mg), age at first assumption, duration of continuous assumption (months), psychiatric disease (yes/no), multiple addiction behavior (yes/no), employment status (employed/unemployed or inactive), and adjusted for age and gender. R = 0.306. R^2^ = 0.093).

### Latent class analysis

**Figure 1:**
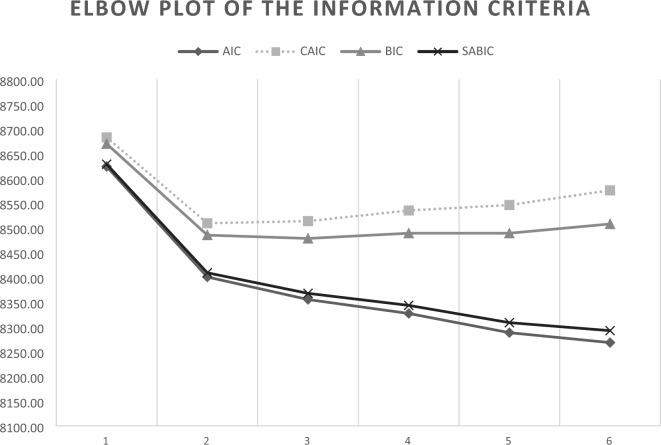
Elbow plot of the information criteria.

Taken as a whole, the 3-class solution showed the better fit (Fig. 1 and Table 4). This solution provided a good level of classification accuracy, with an entropy value of 0.88. These results clearly suggest the high level of classification accuracy of these solutions, with average posterior probabilities of class membership varying from 0.91 to 0.97 (M = 0.934), and with low cross-probabilities ranging from 0.02 to 0.07 (M = 0.04).

**Table 4:** Fit indices for LCA models with 1–6 classes.

Model	LL	#fp	Scaling	AIC	CAIC	BIC	SABIC	Entropy	aLMR	BLRT	AWE	BF
1 class	− 3699.37	13	0.999	7424.74	7481.62	7468.62	7427.43		Na	Na	11,215.93	< 1
2 classes	− 3584.18	24	1.051	7216.36	7321.36	7297.36	7221.31	0.90	< 0.001	< 0.001	10,970.05	< 1
3 classes	− 3552.72	35	1.026	7175.43	7328.57	7293.57	7182.66	0.91	< 0.001	< 0.001	10,975.35	> 30
4 classes	− 3531.67	46	1.111	7155.34	7356.60	7310.60	7164.84	0.83	ns	< 0.001	11,011.90	< 1
5 classes	− 3506.28	57	1.143	7126.57	7375.96	7318.96	7138.34	0.85	ns	< 0.001	11,035.44	< 1
6 classes	− 3487.50	68	1.167	7110.99	7408.51	7340.51	7125.03	0.87	ns	< 0.001	11,078.77	–

*AIC* Akaike information criterion, *AWE* approximate weight of evidence criterion, *CAIC* constant AIC, *BIC* Bayesian Information Criterion, *SABIC* sample adjusted BIC, *BLRT* bootstrap likelihood ratio test.

Concerning the retained 3-class solution, class 1 represents 29.9% of the sample (latent class membership probability = 0.97). Participants in this class had high probabilities of using BDZs as hypnotics (86.5%), of having a borderline risk of QTc prolongation (17.3%) and 10.5% of high risk of QTc prolongation, being former/current abusers of heroin (72.7%), cocaine (85.3%), THC (80.5%) and alcohol (63.2%). Furthermore, individuals in this class showed a long history (M = 139 months) of BDZ abuse, and the highest age of first use of BDZ (M = 33 years old). Thus, this class was labelled as polyabusers with QTc prolongation risk.

Class 2 represents 61.1% of the sample (latent class membership probability = 0.96) and participants in this class had high probabilities of using BDZs as hypnotics (82.9%), 14.6% probability of having borderline risk of QTc prolongation, and 8.6% high risk of QTc prolongation, high probability of not being former/current abusers of heroin (100%), cocaine (92.6%), THC (100%), and alcohol (70.8%). Furthermore, individuals in this class were the youngest (M = 45.1, SE = 0.91), showed the lowest history (M = 96 months) of BDZ abuse, and were 31 years old at their first use of BDZ (M = 31). Thus, this class was labelled as long-time mono-dependence BDZ with residual risk of borderline QTc prolongation.

Class 3 represents 9.0% of the sample (latent class membership probability = 0.91) and participants in this class had high probabilities of using BDZ as hypnotics (60.3%), had 33.6% of probability of having borderline risk of QTc prolongation, and 14.9% of probability of having high risk of QTc prolongation, high probability of not being former/current abusers of heroin (94.6%), cocaine (73.5%), THC (94.8%), and alcohol (50.4%). Furthermore, individuals from this class showed were the oldest (M = 58.6 years old), the lowest age of first use of BDZ (M = 27.5 years old), and the longest history of continuative use of BDZ (M = 317 months). Thus, this class was labelled as long time BDZ abusers with high risk of QTc prolongation.

To test how the classes differed on GGT, AST and ALT levels, the three-step procedure (DU3STEP) was used^58^. Specifically, concerning GGT levels, class 1 showed the highest levels (m = 61.22 U/L. s.e. = 8.93 U/L) that are higher than the cut-off level (> 50 U/L). The other classes showed significant differences, but none were clinically significant. Concerning ALT levels, class 2 (m = 37.52 mU/ml. s.e. = 3.98 mU/ml) showed the highest levels, higher than the cut-off level (> 36 mU/ml). Finally, concerning AST levels, class 2 showed the highest levels (m = 34.63 mU/ml. s.e. = 4.60 mU/ml), higher than the cut-off level (> 33 mU/ml).

## Discussion

Over the past 40 years, BDZ dependence has been on the rise as a public health concern around the world^29^. The present study aims to examine patterns of polysubstance use among a sample of Italian adults with BDZ dependence in relation with QTc prolongation risk.

The Addiction Medicine Unit in Verona treats high-dose BDZ addiction since 2000. To date, there are no papers in scientific literature who treat high-dose BDZ addiction and QTc prolongation risk. Moreover, this cluster analysis brings attention to several clinical aspects. Few studies have examined concurrent and simultaneous patterns of BDZ use and other substance use (e.g., alcohol, stimulants, etc.) by adopting an LCA approach. In general, our results are in line with the literature, finding support for a polysubstance class identification that is similar to, for example, the opioid use class of Votaw and colleagues (2020)^29^ and the high past-month polysubstance use of Ellis and colleagues (2023)^59^. This may suggest that the polysubstance class would be a kind of universal class. However, none of these studies considered BDZ abusers as the target sample.

This study showed a lack of correlation between the QTc interval and the majority of the variables considered, in line with other studies which demonstrating that the QTc interval is only connected to the patients' age and gender^60,61^.

The QT interval does not seem to be affected by non-pharmacological variables such as occupational status and, consequently, by the potential exposure to other occupational risk factors that can affect the QT interval (e.g., shift work, stress)^62^.

Regarding the LCA, it comprises 3 classes. Class 2 is bigger than class 1 and 3, and this data confirms our expectation in accordance with previous studies^30^. It comprises BDZ abusers with low risk of being polyabusers, which are the typical clinical target of our unit. This is an important aspect, because it underlines that BDZ abusers are not only people who have/have had a history of substance misuse. In addition, these patients’ ALT and AST levels are low, which confirms that alcoholic risk is lower in this class than in the other two classes^63^. Class 2 patients have the lowest risk of QTc prolongation among the three patient groups: they are the youngest, have the shortest history of BDZ abuse, and are not current or former users of other drugs. The low likelihood of QTc prolongation can be especially explained by their young age and the lack of polyabuse.

Class 1 is composed by BDZ abusers with a high risk of being polyabusers. It is possible that patients in this class use BDZs to reduce the effect of other substances, in particular cocaine. In this class, BDZs are also used to induce sleep. In this class the risk of QT prolongation risk is higher than in class 2. This could be explained by both their polyabuse of drugs^64,65^, which increases heart risks and reduces quality of life (QoL), and by the patients’ average months of BDZ addiction^66^. Class 1 presents the higher GGT average levels than other classes, confirming the above-mentioned alcoholic risk as GGT levels increase in subjects with an alcohol use disorder^67^.

Class 3 presents a complex clinical situation. This is the smallest class, but it presents several aspects that increase QoL impairment. This class has a lot of similarities with class 1, but their older average age and longest history of continuative use of BDZs could suggests a worsening of the living conditions of these patients. In addition, there is a relevant risk of QT prolongation, so this class presents the highest clinical complexity for the clinician.

The risk of QT interval prolongation is the greatest for patients in this class. Concerning the impact of age on QTc prolongation risk, the elevated risk of QT prolongation in this patient group is most likely caused by their age.

As this study showed no correlation between the duration of the QTc interval and the dosage and duration of BDZ consumption, it is safe to assume that the use of BDZ, even at high dosages, does not constitute a risk for prolongation of the QT interval and the related TdP.

### Limitations of the study

There are several limitations of the present study. First, due to the reduced sample size, our study may have been underpowered to identify differences in BDZ pattern use. Second, this study used cross-sectional data, limiting our ability to determine the temporal sequence among polysubstance patterns. Indeed, our data was retrospectively collected using self-report measures of lifetime substance use. Future studies should consider a longitudinal study design to examine temporal transitions from a class to another class and causal relationships between polysubstance use patterns and QTc. Third, our data were limited to those patients who reported the use of drugs affecting the QTc. In this sense, there is a potential for selection bias as those who did not report this information were not retained for subsequent analyses.

Fourth, the disclosure by participants of a mental health diagnosis was a self-reported information, and an eventual clinical diagnosis was not verified by a trained behavioral clinician nor further verified within patient health records. Finally, participants did not report the frequency and quantity of substance use, nor the sequence of how different substances were used together. Future studies should consider including this information in their measurement to further assess BDZs polysubstance use pattern.

## Conclusions

BDZ addiction is a relevant clinical problem, in particular for high dosage users. The risk of QT prolongation is considered when a clinician sets up a therapy, but this aspect is not very considered for BDZ high-dosage addiction.

This study focuses on three different class of BDZ abusers, with different profiles of QT prolongation risks. Our data confirm the importance of considering QTc aspects especially for female subjects with older age. Moreover, subjects with a high-dose BDZ addiction with low risk to develop addiction comorbidities present a low QT prolongation risk. This data is reassuring for clinicians, since it confirms BDZs’ safety profile. Future study could increase the sample size and the accuracy of LCA.

### Supplementary Information

Supplementary Information.

Supplementary Figure S1.

## Data Availability

All data generated or analysed during this study are included in this published article in a supplementary Excel file.
