# Supplementary material for: High-dose benzodiazepine use and QTc interval prolongation, a latent class analysis study
Source: Sci Rep. 2024 Jan 2;14:155. doi: 10.1038/s41598-023-50489-3 (PMC10762262; doi:10.1038/s41598-023-50489-3)
Supplement: Supplementary file 2 — Supplementary Figure S1. [file 41598_2023_50489_MOESM2_ESM.docx]

Figure 1s. Sample inclusion criteria flow chart.
